# Invariant NKT Cells and Rheumatic Disease: Focus on Primary Sjogren Syndrome

**DOI:** 10.3390/ijms20215435

**Published:** 2019-10-31

**Authors:** Chiara Rizzo, Lidia La Barbera, Marianna Lo Pizzo, Francesco Ciccia, Guido Sireci, Giuliana Guggino

**Affiliations:** 1 Department of Health Promotion, Mother and Child Care, Internal Medicine and Medical Specialties, Rheumatology Section—University of Palermo, Piazza delle Cliniche, 2, 90110 Palermo, Italy; chiararizzo87@gmail.com (C.R.); lidialb90@gmail.com (L.L.B.); 2 Biomedicine, Neuroscience and Advanced Diagnostic—University of Palermo, Via del Vespro 129, 90100 Palermo, Italy; lopizzomarianna@gmail.com (M.L.P.); guido.sireci@unipa.it (G.S.); 3 Department of Precision Medicine—University of Campania “Luigi Vanvitelli”, Via L. De Crecchio 7, 80138 Naples, Italy; francescociccia@tiscali.it

**Keywords:** Sjogren syndrome, iNKT, innate immunity, cytokines, autoimmunity

## Abstract

Primary Sjogren syndrome (pSS) is a complex autoimmune disease mainly affecting salivary and lacrimal glands. Several factors contribute to pSS pathogenesis; in particular, innate immunity seems to play a key role in disease etiology. Invariant natural killer (NK) T cells (iNKT) are a T-cell subset able to recognize glycolipid antigens. Their function remains unclear, but studies have pointed out their ability to modulate the immune system through the promotion of specific cytokine milieu. In this review, we discussed the possible role of iNKT in pSS development, as well as their implications as future markers of disease activity.

## 1. Primary Sjogren Syndrome

Primary Sjogren’s syndrome (pSS) is an autoimmune disease characterized by chronic inflammation of exocrine glands. In particular, salivary and lacrimal glands are the main target in pSS disease, characterized by a progressive lymphocytic infiltration causing the hallmarks symptoms of pSS: xerophthalmia and xerostomia [1].

The extraglandular manifestations (arthritis/arthralgias, vasculitis, and neuropathy affecting mainly peripheral nerves) can even occur in pSS underlining the systemic involvement of the disease [2].

Moreover, patients suffering from pSS are at high risk of developing B-cell non-Hodgkin’s lymphoma as a severe complication of the disease. It is estimated that approximately 5% of patients can develop non-Hodgkin lymphoma, and the risk is considered 7- to 19-fold higher when pSS patients are compared to the general population [3]. The most frequently identified type of lymphoma affects MALT (mucosal-associated lymphoid tissue) in salivary glands; however, more aggressive subtypes of lymphomas as large diffuse B-cell variants have been described [4]. Tobon et al. showed that Fms-like tyrosine kinase 3 ligand (Flt-3L) might be associated with lymphoma in pSS, suggesting its role as a biomarker to identify patients at high risk to develop proliferative disease [5]. Interestingly, in studies focused on salivary gland histology, another possible predictor of lymphoma development was identified. Low miRNA200b-5p levels were identified in pre-lymphoma pSS patients several years before the onset of hematological disease [6]. The prevalence of pSS was about 0.5% with a mean onset age in the 4th–5th decade, even if a later onset between the 6th–7th decade was quite common. Like other autoimmune diseases, pSS affects prevalently women with a female to male ratio of 9:1 [7].

pSS pathogenesis appears to be multifactorial: immune system dysregulation, genetic, epigenetic, environmental, infectious, and hormonal factors could play a role in the complex etiology of the disease. Several chemokines and cytokines account for both an inflammatory (CXCL13 CXCL10, CXCL8, CCL2, IL-10, and IL-6) and a pro-fibrotic (CXCL14, CCL28, tumor necrosis factor-related apoptosis-inducing ligand, and TGF β) effect. This intricate milieu finally triggers an abnormal immune response involving both innate and adaptive immune cells, leading to an initial process of epithelitis. In target tissues, the presence of ectopic lymphoid structures can be easily identified with evidence of B-lymphocyte expansion and the production of autoantibodies. This confirms the key role of adaptive autoimmunity in pSS at least in advanced phases of the disease [8].

To date, the search for reliable biomarkers able to ameliorate the diagnostic algorithm and the prognostic stratification of pSS patients is a major topic in pSS and remains an unmet need.

Autoantibodies, anti-Ro e anti-La, are classically considered hallmarks of pSS. However, new autoantibodies, known as tissue-specific antibodies (TSAs), are emerging as possible new biomarkers. TSAs include anti-salivary protein 1 (SP1) and anti-carbonic anhydrase 6 (CA6) that are associated with early stages of pSS and worse ocular manifestations, and anti-calponin antibodies are more related to peripheral neuropathy. Larger studies are needed to confirm the utility of these antibodies in routine clinical practice [9,10]. Considering pathogenesis, interferon (IFN) type I seems to be a central actor in pSS. It induces a wide range of genes involved in pro-inflammatory immune response and correlates with a more severe disease course [11]. This overexpression of IFN-related genes was described in both blood and salivary glands of pSS patients, characterizing the so-called IFN-signature in this specific rheumatic disease. Thus, IFN type I and its surrogates, as myxovirus-resistance protein A or the identification of IFN-induced genes, may represent possible biomarkers of pSS [12,13]. Among chemokines, CXCL13, which can be detected in peripheral blood as well as in salivary gland tissue, was demonstrated to correlate with disease activity and lymphoma development. This observation makes CXCL13 an attractive biomarker to monitor pSS progression [14]. 

Screening the non-invasive biomarkers from the saliva and tears has a significant potential too. These fluids represent a crucial source of valuable biomarkers as LACTO or LIPOC-1 tear proteins or S100A8/A9, a molecule found in the saliva of pSS that correlates with a higher risk of developing lymphoma [15,16].

Even the imaging techniques are considered future possible biomarkers of disease. Salivary glands ultrasonography stands out as the most promising field, and great research efforts are currently ongoing to define a clear possible role of this technique in pSS diagnosis, follow-up, and response to treatment. Particularly, elastosonography, which has the ability to measure the degree of glandular elasticity, seems to be a reliable tool to identify pSS patients with high sensitivity, specificity, and negative predictive value [17].

In recent years, great attention has been directed towards innate immunity, its role in the early stages of pSS, and its interplay with adaptive immunity. These cells, including dendritic cells, macrophages, salivary gland epithelial cells, and natural killers (NK), act as a first-line defense against exogenous and endogenous molecules. Their dysfunction contributes to initiating the inflammatory process in exocrine glands [18]. Up to date, the specific role of innate immunity in pSS remains poorly understood, and further research in this intriguing field is required. However, a growing body of evidence concerning autoimmune disorders has focused attention on a specific subset of cells, named NKT (natural killer T cells), that works as a bridge between adaptive and innate immunity [19]. They have been described in several autoimmune diseases. In particular, in rheumatology, their role in systemic lupus erythematosus (SLE), rheumatoid arthritis (RA), and spondyloarthritis (SpA) has been investigated. Poor literature describing their role in pSS exists, and in this review, we have focused attention on data available regarding NKT cells, particularly, invariant NKT cells (iNKT), and pSS pathogenesis [20].

## 2. Invariant NKT Cells

Natural killer T cells (NKT) are a unique population of lymphocytes characterized by sharing the feature of both T cells and natural killers. These cells differentiate from thymic precursors and are able to recognize both selves (e.g., isoglobotrihexosylceramide (iGb3)) and non-self-lipid antigens (e.g., α-glycuronylceramides detected in the cell wall of Gram-negative bacteria) [21]. NKT cells have been studied in a wide range of disease conditions involving autoimmunity, infections, allergy, cancer, and transplant medicine. Mature NKT cells can produce IFN-γ and massive amounts of IL-4, this specific feature is distinctive of this cell subset and differentiate them from other lymphocytes [19]. 

Three subsets of NKT have been described so far. Type I NKT, also known as invariant NKT (iNKT), type II NKT, and NKT-like cells. They differ in the expression of specific antigen receptors (TCR, T cell receptor) [22].

Invariant NKT cells are identified by the expression of a specific semi-invariant T cell receptor (TCR) and natural killer receptors, such as CD161 [23]. The iNKT TCR consists of a single α-chain paired with a specific set of Vβ-chains, Vα24Jα18Vβ11, that recognizes glycolipids when associated with a glycoprotein belonging to the CD1 family named CD1d, which is expressed on antigen-presenting cells (APC) [24]. The complex, CD1d-glycolipids, strongly activates NKT, leading to cytokine and chemokine production. In vitro activation of iNKT via glycolipid stimulation is obtained using a synthetic antigen derived from a marine sponge called α-galactosylceramide (α-GalCer). This antigen shares a common structure with the microbial α-glycuronylceramides found in the Gram-negative cell wall [25]. Once activated, iNKT can polarize their cytokine profile towards both type 1 T-helper cell-like or type 2 T-helper cell-like response [26].

iNKT include CD8^+^, CD4^+^, and double-negative CD4^-^/CD8^-^ cells. Each of these subsets produces a different group of cytokines, as previously mentioned, accounting for either a protective or a pathogenetic role in the process beneath autoimmune diseases [27].

In an elegant study by Yang et al., iNKT cells activated via α-GalCer demonstrated to reduce autoantibody production, as well as IL-10 secretion determining a tolerogenic environment. In particular, CD1d seems to play a pivotal role in their activation. CD1d^+^ B-cells are able to induce iNKT activation, leading to a decrease in autoreactivity; in contrast, CD1d deficiency determines an increased release of autoantibodies by B-cells exposed to activated iNKT. The role of CD1d was further stressed by the observation that activated iNKT co-cultured with wild type or CD1d deficient B-cells-reduced autoantibody production without affecting total IgG levels. This demonstrates that the regulatory role of iNKT on autoimmunity strongly correlates with CD1d expression on B-cells, and CD1d is upregulated in autoreactive B-cells. Interestingly, several reports have shown how CD1d deficient experimental models develop severe spontaneous or induced SLE. In conclusion, it seems important to stress that iNKT cells suppress autoreactivity without causing a global depletion of B-cells function. This observation highlights the importance of focusing future research on this specific cell subset, clarifying its implication in rheumatic disease to use iNKT as possible therapeutic agents in the future [28].

Interestingly, in infectious disease, NKT cells seem to have a major role when Gram-negative pathogens are implicated. In particular, an important contribution of NKT was demonstrated in the clearance of Gram-negative, LPS negative bacteria as Sphingomonas, a microbe that can cause severe disease in immunocompromised patients, or Ehrlichia, responsible for tick-borne infections [29]. In CD1d- or Jα18-deficient experimental models, a defective activation of NKT was observed, and consequently, impaired clearance of Ehrlichia was evidenced [30]. On the other hand, Gram-negative, LPS positive bacteria appeared to trigger an autoreactive NKT activation. LPS recognized via TLR in APC causes the release of IL-12 and the exposure on the cell membrane of endogenous lipids, as iGB3, that stimulates NKT cells [31]. Both exogenous and endogenous glycolipid antigens activate NKT cells during microbial infections [19]. 

An intriguing link between infections, NKT, and autoimmune disease development was described in primary biliary cirrhosis (PBC). The serological hallmark of PBC is the presence of antimitochondrial antibodies. Their target is commonly found in a strain of Sphingomonas. A study by Selmi et al. in 2003 evidenced that PBC patients that presented Sphingomonas in stool samples were even seropositive to Sphingomonas.

Moreover, in PBC, NKT was found to be abundant in liver specimens while reduced in peripheral blood. This observation suggested that an aberrant response to Sphingomonas driven by NKT cells could be responsible for the inflammatory process leading to cirrhosis in PBC [32]. It seems important to stress that PBC can be specifically associated with pSS, suggesting a more complex systemic involvement. A common background between different faces of these autoimmune diseases exists, and iNKT is emerging as a possible key player in this intricate process.

## 3. iNKT and Their Controversial Role in Rheumatic Disease

The harmful versus the protective role of iNKT cells in rheumatic disease has been the subject of extensive research, particularly in autoimmune diseases, such as SLE, RA, and Sjogren’s Syndrome (SS) [20].

The possibility that the exposure time of iNKT to antigens can activate an anti-inflammatory versus a pro-inflammatory cytokine production panel was highlighted. Specifically, this time-dependent model points out how a short-term activation (2–4 h) determines a prevalent secretion of molecules, such as IL-10, that act regulating the immune system. On the other hand, prolonged exposure to ligands (>6 h) causes a marked increase in IFN-γ, which drives an inflammatory destructive response [33]. This possible different activation of iNKT could also be related to environmental factors.

Nowadays, however, more studies are required to clarify the role and the potential effects of iNKT in rheumatic disease. An important contribution could derive from studying these cells in pSS. Most studies have focused attention on the number of iNKT among peripheral blood mononuclear cells (PBMC), while few papers have investigated their presence in target tissues. This is mainly due to the difficulties in obtaining specimens of anatomical sites of inflammation. In this regard, pSS represents a good autoimmune disease model to study the compartmentalization of iNKT cells as biopsies of salivary glands are routinely and easily performed [34].

### 3.1. iNKT in SLE

In this regard, SLE can be considered a complex model to assess both the immune-potentiating and suppressive effects of iNKT [35]. In SLE animal models, dichotomic results since the early 2000s have been described. This pointed out that genetics plays an important role in driving iNKT response in different cytokine milieu. For example, CD1d deficiency was linked to worsening lupus nephritis in an SLE mouse model induced by hydrocarbon oil pristine, and the same alteration seemed to determine severe skin manifestation in MRL-lpr mouse model [36]. However, these specific results on skin manifestations were not confirmed in MRL-lpr models strains. Considering lupus nephritis, exogenous stimulation with α-GalCer in MRL-lpr mice and NZBxNZW F1 mice obtained opposite effects. In the first mouse model, no effect on the renal disease was detected, while in the second one, a worsening in renal manifestation was observed. In particular, the stimulation with external antigens produced a marked expansion and activation of iNKT. These cells were then transferred in healthy mice, causing nephritis, thus clarifying the potentially harmful role of activated iNKT [37,38,39,40].

In other murine models, the administration of α-GalCer was performed to test its effect on proteinuria. Specifically, in BALB/c mice, the stimulation of iNKT determined a decrease in proteinuria level, while the opposite effect was observed in SJL models. This dichotomic result was further investigated in a study published later, which highlighted how iNKT did not produce IL-4 in SJL mice, as observed in BALB/c models. Once again, results confirmed the importance of the genetic background in the activation of iNKT [41].

The protective role of iNKT in SLE has been linked to their ability to regulate IgG production; type I NKT can suppress autoreactive CD1d+ B cells preventing autoantibodies release [42]. To support this evidence, several studies have pointed out that iNKT cells are reduced in SLE patients and first-degree relatives. This could be a heritable trait involved in the complexity of SLE pathogenesis [35]. Moreover, in an SLE experimental model, a short-term stimulation with α-GalCer demonstrated an increase of IL-10 that prevented autoantibody secretion, determining a tolerogenic cytokine environment [43].

### 3.2. iNKT in RA

In RA, the role of iNKT was also described. Particularly, the reduction of circulating CD4+ and CD4- iNKT cells determines the lack of protection from the inflammatory joint disease [44]. Additionally, in joint tissue, a decrease in number and impaired function of type I NKT were highlighted [45].

However, as described in experimental models of SLE, a dichotomous function of iNKT has been reported. iNKT has been shown to promote several pathways of differentiation of Th cells, including Th1, Th2, Th17, and Treg [46]. It seems to depend on the cytokine milieu and on the kind of arthritis model studied on which iNKT act. In collagen-induced arthritis (CIA), the administration of exogenous α-GalCer to activate iNKT determined the development towards a protective cytokine pathway, promoting a Th2 environment [47]. Similar results were obtained stimulating iNKT with OCH, a sphingosine-truncated analog of α-GalCer, that determined suppression of CIA development. However, a time-dependent and ligand-dependent response emerged from the studies carried on [48]. In particular, exogenous stimulation of iNKT with different ligands promoted an increase in IFN-γ in the early phases of the disease, determining arthritis progression and severity, while in the later stages of RA, the presence of the same molecule appeared to protect from arthritis damage [49,50]. In contrast with this evidence, in other experimental models, the lack of CD1d or iNKT cells appeared to protect against the progression of inflammatory joint disease. For example, in a model of arthritis obtained with anti-collagen type II monoclonal antibodies, the lack of iNKT was associated with an important reduction in disease severity; on the other hand, iNKT cells activation in the same models worsened joint inflammation [51].

These controversial results stress the fact that phenotypical differences in iNKT in mice and humans are important and could partially account for the differences highlighted in different studies [52,53].

### 3.3. iNKT in SpA

In SpA, the role of iNKT is still very mysterious. However, several studies have focused attention on this cell subset because of a possible protective role in gut infection and autoimmune disease as Crohn or ulcerative rettocolitis [54,55]. Gut and, in particular, intestinal microbiome appear to be involved in SpA pathogenesis [56,57]. In a study by Jacques et al., a murine model of SpA lacking iNKT was obtained, and the progression of arthritis was compared to classic SpA models. In iNKT deficient models, articular damage, as well as intestinal inflammation, progressed more rapidly and showed more severe features, including bone erosions, bone marrow invasion, and inflammation at sacroiliac joints. In addition, ileal damage was characterized by more important inflammatory infiltration and consequent villous destruction. To confirm this observation, authors transferred activated iNKT in iNKT lacking mice before the onset of articular symptoms. After disease onset, a deceleration of disease progression was observed in these models when compared to mice that did not receive iNKT cells [58].

Moreover, iNKT activation was related to high TNF level and long exposure to dendritic cells (DC). TNF may act by determining an upregulation of CD1d in DC that activates iNKT, favoring their immunoregulatory activity. The SpA is known to present a high level of TNF, and maybe iNKT can be involved in the pathogenesis of this rheumatic disease. iNKT can differentiate under inflammatory conditions and can migrate at mucosal sites [59,60], as evidenced in experiments with germ-free mice where iNKT cells underwent further maturation in the intestinal mucosa. It has been hypothesized that DC presenting intestinal derived microbial antigens can trigger iNKT activation. Interestingly, DC was detected in a high percentage in synovial fluid from SpA patients [60]. More research on iNKT and their involvement in SpA pathogenesis is required, and interesting results can be derived even from studying these cells in inflammatory bowel disease.

Nowadays, more studies are required to clarify the role and the potential effects of iNKT in rheumatic disease. An important contribution could be derived from studying these cells in pSS. Most studies have focused attention on the number of iNKT among peripheral blood mononuclear cells (PBMC), while few papers have investigated their presence in target tissues. This is mainly due to the difficulties in obtaining specimens of anatomical sites of inflammation. In this regard, pSS represents a good autoimmune disease model to study the compartmentalization of iNKT cells as biopsies of salivary glands are routinely and easily performed [61].

### 3.4. iNKT in Systemic Sclerosis (SSc)

In SSc, a possible role of NKT has been suggested in a study published in 2005. Researchers demonstrated, in a sample of 50 SSc patients, that NKT cells were reduced among peripheral cells. Moreover, their number inversely correlated with markers of disease activity, such as erythrocyte sedimentation rate, levels of circulating gamma-globulins, and serum concentration of IgG. Probably NKT absence or impairment may contribute to the downregulation of the physiological immune response, inducing autoreactivity that is involved in SSc pathogenesis [62]. However, there is no sufficient available literature on this specific topic to better understand how iNKT cells may interfere with the process beneath SSc development and progression. In this rheumatic disease, another cell group known as mucosal-associated invariant T (MAIT) cells, which share some common features with iNKT, has been studied. This observation, once again, underlines the importance of the innate immune system even in SSc [63].

## 4. iNKT in Sjogren Syndrome

Recent evidence on pSS pathogenesis underlined the importance of iNKT in the process leading to antibodies production [61]. To the best of our knowledge, very few papers have focused attention on these cells and pSS etiology.

A rapid communication published in 2001 evidenced a decreased number of iNKT among patients suffering from autoimmune diseases, including pSS. A possible relation between low iNKT percentage and autoreactive tissue damage was addressed. The number of iNKT cells was proven to be significantly reduced in pSS, as well as in RA and SLE, while it was normal in diseases that commonly do not imply structural damages as myasthenia gravis and Graves disease. As a limit, only circulating iNKT was evaluated without analyzing tissue specimens of salivary glands [44]. 

Nevertheless, iNKT was also observed in pSS salivary gland tissues using CD3+CD16+ and CD56+ as markers to identify these cells. However, according to literature, these markers are not sufficient to identify iNKT [64,65]. After several years, another paper aimed to assess the frequency and function of iNKT in peripheral blood and salivary glands tissue from pSS patients, trying to identify iNKT cells using a tetramer construct. In detail, α-GalCer associated with a specific probe (CD1d/α-GalCer) was used to interact and bind specifically the invariant TCR of iNKT [64]. At the tissue level, iNKT was undetectable, and a high number of autoreactive B cells were identified. So, the different evaluation could be due to a technical difference in iNKT detection.

**Figure 1:**
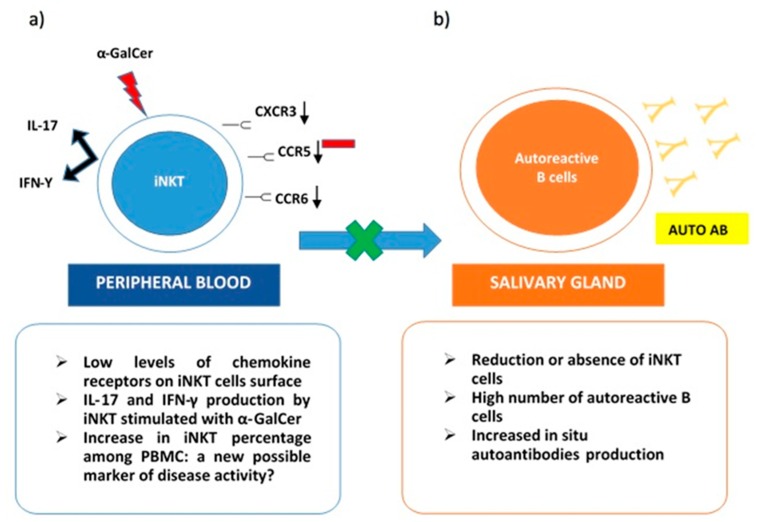
iNKT cells in Sjogren Syndrome. (**a**) in peripheral blood iNKT express low levels of chemokine receptors (CXCR3, CCR6, CCR5) on their surface. This could explain their reduced presence in salivary glands due to impaired migration. iNKT from pSS peripheral blood samples stimulated with a-GalCer produce both IL-17 and IFN-γ; (**b**) in the salivary gland, reduction or absence of iNKT cells determines the loss of their role as negative regulators on autoreactive B cells and the consequent in situ production of autoantibodies.

On the other hand, iNKT was expanded in peripheral blood samples from pSS patients. These cells stimulated with α-GalCer produced both IL-17 and IFN-γ. Interestingly, low levels of chemokine receptors as CXCR3, CCR6, and CCR5 were detected on the iNKT cells’ surface. This evidence may justify the impairment of activated iNKT to migrate towards inflammatory tissues. Specifically, in pSS, their absence in immune cells infiltrates in salivary glands can explain the lack of control on autoantibodies in situ production (Figure 1). The co-culture of B cells from pSS salivary glands with iNKT demonstrated that anti-SSA production was reduced in the presence of activated iNKT. This inhibitory activity on autoreactive B cells correlated with the number of iNKT in cultures [64]. The scientific background was mainly derived from the previously described results obtained in SLE. In particular, a reduction or the absence of iNKT in this autoimmune rheumatic disease was related to an increase in autoantibodies production, confirming the protective role of iNKT as negative regulators of autoantibodies producing B cells [66]. Taking into account the ability of iNKT to perform their regulatory function on self-reactive B cells, safeguarding non-self-reactive B cells [28], a possible future role, as a valid therapeutic target for iNKT emerges.

However, a deeper characterization of iNKT cytokine/chemokine profile and their surface receptors assessment, as well as of their migratory pattern, is required to identify a potential therapeutic option based on iNKT.

In addition to this, another strength point is the increased percentage of iNKT among PBMC in pSS. The number of NKT cells was found higher in pSS patients who had extraglandular manifestation, while the amount of NKT cells in patients without systemic involvement and controls was similar. Such a result could be due to an extreme attempt to counter-regulate an important ongoing inflammatory process. Furthermore, a significant increase in T regulatory type 1 (Tr1) cells and CD4^+^ CD25^+^ Treg cells was observed in patients with pSS with extraglandular manifestations compared to patients without these features. This data would seem to further confirm the presence of a possible compensatory mechanism, especially in the more severe forms of pSS with systemic inflammatory status [67].

It is well known that in pSS, there is a lack of markers of disease activity, and the evaluation of iNKT in peripheral blood could become an effective marker to assess it [64].

On the other hand, other studies investigated the presence of iNKT only among PBMC, obtaining, once again, the conflicting results. Several studies found a decrease in the number of iNKT in peripheral blood samples of pSS patients, while other researchers determined that the same cells were increased in pSS. In particular, Sudzuis et al. showed a significant reduction in the absolute NKT cell count in peripheral blood of patients with seropositive pSS, possibly ascribed to genetically determined lymphopenia. Furthermore, it has been hypothesized that this reduction could be due to migration in inflammatory sites or to apoptosis [68]. 

Moreover, two different groups of patients: responder and non-responder to α-GalCer in vitro stimulation were studied. In responder patients, a possible cause of iNKT global reduction could be an insufficient amount of natural ligands on APC. On the other hand, possible causes of iNKT unresponsiveness could be related to a specific dysfunction of iNKT or their overstimulation in a chronic inflammatory autoimmune environment. In this case, their prolonged activation could lead to a selective decrease in their number [24].

In addition to this, a paper published in 2017 by Davies et al. tried to better characterize peripheral leukocyte population of patients with pSS. Patients were further stratified according to the presence or absence of autoantibodies SSA/SSB and extraglandular manifestations. Researchers evidenced that the pSS patients, in particular, if seropositive and affected by systemic symptoms, presented a significant decrease in lymphocyte subpopulation when compared to controls. Conversely, the same patients appeared to have a higher number of granulocyte and monocyte subpopulations. Taking into account iNKT cells, an increase in this specific subgroup was found out in patients affected by a milder disease without important extraglandular involvement [69]. This interesting result, once again, confirms a possible protective role of NKT cells in the disease process leading to pSS. 

In conclusion, it seems important to underline that different clusters of disease exist, and complete characterization of patients, stratified considering cellular and cytokine/chemokine profile, is mandatory to better understand disease phenotypes and subsequently to approach a specific targeted therapy [70].

## 5. Conclusions and Future Perspectives

It comes out clearly that extensive research on the role of iNKT in pSS pathogenesis is required. A better understanding of how innate and adaptive immunities contribute to disease development will grant the possibility to find out new therapeutic targets [71].

In this regard, the role of iNKT and their possible involvement seems a promising research field in pSS [72]. 

Up to date, the majority of research has focused attention on iNKT cells among PBMC, and there is a lack of studies analyzing this subgroup of cells in salivary gland tissue. Moreover, deeper comprehension of mechanisms involved in the cytokine/chemokine profile of iNKT is needed, as well as a better description of their migratory pattern and role in bridging innate and adaptive immunity [73]. First reports at the beginning of the century described heterogeneous groups of cells, and still, a lot of work is required to better define the different subsets and their activity not only in autoimmune disease but also in cancer/tumor surveillance and infectious diseases [74].

Even technical advancement in determining and correctly identifying iNKT cytokine production is a current topic of research. Sag et al. described a new technique to remove dead and apoptotic cells to better identify specific cells’ subsets as IL-10 iNKT, as well as other molecules usually more difficult to quantify like IL-2, IL-10, IL-17A, and GM-CSF [75].

Future perspectives will include the possible use of iNKT as a specific therapy for rheumatic disease as pSS or even as predictors to respond to treatment. In preclinical studies, the therapeutic efficacy of iNKT in suppressing autoimmunity was evidenced; most papers focused attention on autoimmune diseases, such as SLE and RA. In particular, the stimulation of iNKT cells with glycolipid antigens was shown to shape their immune response towards a Th1 or Th2 cytokine profile. Th2 responses correlate with disease protection, whereas Th1 is related to disease exacerbation. Analogs of natural glycosphingolipids were used to stimulate the iNKT act, as mentioned above, in a time-dependent way, enhancing a protective cytokine production. Other variables influencing iNKT response are the frequency of exposure to antigens, their interaction with the gut microbiome, as well as the specific milieu they are in. According to this, differences can be evidenced in the various experimental model strains used to study iNKT activity. KRN7000 is a synthetic version of αGalCer used to stimulate iNKT cells; its analogs can differently activate iNKT. For example, OCH is considered a more Th2 inducing analog, which promotes a Th2 profile on iNKT, determining an increase in IL-4, IL-10, and IL-13 production. These cytokines act directly, inducing a tolerogenic environment, and indirectly, activating immunoregulatory cells able to further promote tolerance. Among these cells, Foxp3^+^ Tregs and immune-suppressive myeloid cells as MDSCs, dendritic cells, macrophage, and neutrophils seem to play an important role in the suppression of autoreactive cells. 

On the other hand, KRN7000 analog αGALCER was shown to shape iNKT cells’ response toward a Th1 profile, mainly characterized by a high amount of IFNγ. This specific milieu inhibits autoimmunity suppressing pathogenetic T cells, as Th17 and Th1, inducing their energy.

Taking into account the whole evidence from experimental models, the administration of KRN7000 and its analogs appears a promising new therapeutic approach for autoimmune diseases. [76,77]. Using iNKT as a therapeutic agent in rheumatology has yet been suggested in systemic sclerosis. Researchers demonstrated that these cells presented an impaired activation and a global reduction in number in systemic sclerosis patients independently from the disease pattern they have. In the future, subsequently transferring the culturing and expanding in vitro iNKT cells to patients could be a promising therapeutic approach in systemic sclerosis [78].

The same goals are a need in pSS, a disease that still lacks specific therapies designed to interfere with the biological mechanisms involved in the disruption of the immune system. In this regard, interesting results emerge from clinical trials aimed to assess biologic treatment in pSS. Rituximab and belimumab target B-cells activity, and rituximab was proven to determine a decrease even in Th17 cells that are considered important effectors of chronic inflammation in pSS [79]. Interestingly, rituximab induces a significant reduction of IL-22 expression in the salivary glands of pSS and determines an amelioration on both whole saliva flow rate and lacrimal gland function, suggesting that IL-22 modulation could partly account for the efficacy of anti-CD20 treatment in pSS [80]. Several trials, including these therapeutic agents, are currently ongoing. Other promising fields are costimulatory molecule abatacept, kinase inhibitors, IL-6 targeting agents as tocilizumab and anti-TNFα agents. However, still, a lot of research is needed to clarify biologic mechanisms that lead to pSS development and progression [81]. As previously highlighted, the possibility of having tissue specimens in pSS can help in stratifying patients according to specific histopathology. Furthermore, the efficacy of specific therapies can be evidenced, studying the modification of salivary gland tissue pre and after treatment. This implies the optimal standardization of salivary gland histology to determine the degree of disease activity [82]. In this regard, the identification of iNKT cells among cell infiltrates in tissue samples or peripheral blood can help in obtaining new markers of disease.

In conclusion, iNKT stands out as a promising research field to obtain new information on pSS etiology, as well as to grant newer therapies to patients. Further elucidation of their activity in suppressing autoantibodies production and on their interaction with B-cells will help to determine a possible role for iNKT cell-based therapies for autoimmune disease. The limited polymorphism of CD1 [83] genes makes this subset of cells particularly appealing as therapeutic agents as it overcomes problems classically related to therapies targeting MHC I and II systems [28].
